# Association of height, foot length, and pubertal development in children aged 3–18: a cross-sectional survey in China

**DOI:** 10.3389/fpubh.2024.1322333

**Published:** 2024-02-12

**Authors:** Hua-Hong Wu, Ya-Qin Zhang, Cheng-Dong Yu, Li Yang, Chen Tao, Wen Shu, Tao Li, Guimin Huang, Dongqing Hou, Fang-Fang Chen, Jun-Ting Liu, Shao-li Li, Xin-Nan Zong

**Affiliations:** Capital Institute of Pediatrics, Beijing, China

**Keywords:** foot length, height, Tanner stage, children, pubertal development

## Abstract

**Objective:**

This study aimed to analyze the growth patterns of height and foot length (FL) among Chinese children aged 3–18 and examine their associations with puberty development.

**Methods:**

A cross-sectional survey was conducted in September 2022 in Beijing. Data were collected through questionnaires and on-site physical examinations. The growth patterns and velocity of height and FL in different age groups were described, and their associations with puberty development were analyzed.

**Results:**

From an age perspective, the peak FL growth occurred between 9 and 11 years (boys were 11 years and girls were 9 years), while the peak height growth occurred at 11 ~ 13 years for boys and 9 ~ 11 years for girls. Additionally, boys and girls reached 99.0% of their final FL at the ages of 14 and 13, respectively, while they reached 99.0% of their final height at the ages of 16 and 15, respectively. From the perspective of Tanner stage, the age of peak FL growth in boys coincided with the age of the G_2_ stage, while in girls it occurred slightly earlier than the mean age of the B_2_ stage. The peak height growth for both boys and girls occurred between Tanner stages 2 and 3.

**Conclusion:**

Boys and girls reach their peak FL growth at 11 and 9 years old, respectively, which were both 2 years earlier than their peak height growth. The peak FL growth occurred around the onset of puberty, while the peak height growth occurred between Tanner stages 2 and 3.

## Introduction

The growth and development patterns of children vary considerably across different races and genders. Even within the same individual, different parts of the body develop at distinctive velocities (1). In terms of physical growth, Tanner proposed a growth gradient from distal to proximal in children, where peak growth of foot length (FL) may precede peak growth of other parts of the body such as the limb bones and spine. Peak FL growth may be an early sign of puberty (2). Parents can use a child’s FL or shoe size as indicators to monitor their development stage, promptly identify potential issues in the growth process, and provide timely intervention.

Nevertheless, due to the disparities in children’s developmental patterns from different countries, previous studies on the age of peak FL growth have shown significant variations ranging from 8 to 13 years for boys and from 7 to 11 years for girls, and there may not be just one peak for FL growth (1, 3–5). A Spain survey showed that the age of peak FL growth coincided with the age of Tanner 2 (4). In China, studies in this field have been relatively scarce. The most recent study in 2018 suggested that there may be more than one peak in FL growth for children aged 7–12 years. However, due to the age range of their sample (some subjects may have not entered puberty at the age of 12), the study lacked data on puberty development, making it less significant for monitoring puberty growth and development spurts (6). Furthermore, in recent years, with the observable trend of early puberty accompanied by an early growth spurt in Chinese children (7), there has been no research exploring the association between the peak growth of FL and height and their potential connection with the puberty process.

This study aimed to fill this gap by analyzing data from children aged 3–18 years, including preschool, school age, and until the end of puberty. Our focus was on exploring the contemporary FL and height growth patterns of Chinese children, finding the ages of peak FL growth and peak height growth, as well as their associations with puberty development. This information could potentially contribute valuable data for physicians, healthcare professionals, and parents in regular monitoring and related studies.

## Materials and methods

### Study design

This study was conducted using a cross-sectional survey method in September 2022 in Beijing. Using the random cluster sampling method, a total of nine kindergartens, primary schools, and middle schools in the Tong Zhou district of Beijing were randomly selected, and 3,696 children aged 3–18 years were included in the study, except for those who were unable to participate in physical examinations due to trauma or physical discomfort. The study protocol was reviewed and approved by the Ethics Committee of the Capital Institute of Pediatrics (No: SHERLL2022043), and informed consent was obtained from the guardians and/or students.

### Methods

#### Age and age groups

The age of the children was calculated based on their birth date and the survey data obtained through a questionnaire. The age groups were divided into 1-year intervals, such as the 3~ age group with children between 3 years old and 1 day before their 4th birthday; the 4~ age group with children between 4 years old and 1 day before their 5th birthday; and so on. The 17 ~ 18 age group comprised children between 17 years old and 1 day before their 18th birthday. A total of 15 age groups were included.

#### Height

The height was measured by trained staff using a mechanical stadiometer (Harpenden Portable Stadiometer, UK). All children were asked to remove their shoes, socks, hats, and outerwear and stand upright in a normal posture with their heels, buttocks, and shoulder blades touching the column at the same time while keeping their heads in an upright position. The data were recorded in centimeters (cm) and rounded to one decimal place.

#### FL

The study subjects were required to remove their socks and stand in an upright position with their weight evenly distributed. FL was measured using the Brannock device (junior model, Syracuse, New York, United States), which measured the longest distance from the heel to the toe of the right foot, expressed in centimeters (cm) accurate to one decimal place.

#### Growth patterns and velocity

The growth pattern and velocity of height and FL vary significantly, making direct comparisons of actual measurements unsuitable. To address this, we need to convert the actual values of height and FL into percentages. The method was to calculate the percentages of height and FL in different age groups for the final height and final foot length. These percentage values can be directly compared to describe the different growth patterns of height and FL. Additionally, the increase in percentage values between adjacent age groups (Δ %) is calculated to describe the growth velocity (GV) for each year. The Δ% in different age groups reflects changes in GV, and the age of the peak growth can be identified.

#### Pubertal development

According to the Tanner stage, boys were divided into five stages (G1–G5) based on the degree of penis development, and girls were also divided into five stages (B1–B5) based on breast development. The onset of puberty is marked by boys reaching stage G2 and girls reaching stage B2.

### Statistical analysis

The data were analyzed using the SPSS Statistics statistical program version 22 for Windows 10 (IBM Corp., Armonk, New York, United States). The quantitative data were expressed as χ ± SD, and the distribution of the 10th, 50th, and 90th percentile values of height and FL for each age group was presented. The standard deviation scores (SDSs) for height, weight, and BMI were calculated using the LMS method as HtSDS, WtSDS, and BMISDS, based on the growth reference for Chinese children aged 0–18 years (8, 9). The *t*-test was used for intergroup comparison of quantitative data, and a *p*-value < 0.05 was considered statistically significant. The percentage of height and FL for each age group was calculated as the value of height or FL in that age group divided by the height or FL of the 17 ~ 18 year group, expressed as percentages. We also focused on the age of height, and FL reached 99% of their final values, which reflects the age of near final height or final FL. The GV was calculated as the percentage value of the latter age group minus the percentage value of the former age group (Δ %).

## Results

### General information

This study enrolled a total of 3,696 children aged 3–18 years. After excluding 206 children without FL data, 3,490 children were included in the data analysis. Of these, 1742 were boys (49.9%) and 1748 were girls (50.1%). The HtSDS, WtSDS, and BMISDS of all children were 0.32 ± 1.04, 0.72 ± 1.54, and 0.71 ± 1.58, respectively. Table 1 presents the comparison of age and physical growth levels between boys and girls. It was noteworthy that boys had higher height, weight, BMI, and FL than girls (*p* < 0.001), but there were no significant differences in HtSDS, WtSDS, or BMISDS between them.

**Table 1:** Comparison of age and physical growth levels between boys and girls.

Variable	Boys	Girls	*t*	*p*
*N* (%)	1,742 (49.9)	1,748 (50.1)		
Age (y)	11.6 ± 3.6	11.9 ± 3.5	−2.228	0.026
Height (cm)	152.9 ± 21.8	149.4 ± 17.4	5.143	<0.001
Weight (kg)	51.9 ± 23.7	46.6 ± 17.8	7.512	<0.001
FL (cm)	22.9 ± 3.4	21.7 ± 2.5	11.725	<0.001
BMI (kg/m^2^)	21.0 ± 5.5	20.1 ± 4.8	5.367	<0.001
HtSDS	0.30 ± 1.05	0.34 ± 1.03	0.032	0.975
WtSDS	0.72 ± 1.53	0.71 ± 1.51	0.581	0.561
BMISDS	0.74 ± 1.56	0.68 ± 1.53	1.111	0.266

FL is foot length. BMI is the body mass index, calculated as weight (kg) divided by height squared (m^2^). HtSDS is the standard deviation score for height, WtSDS is the standard deviation score for weight, and BMISDS is the standard deviation score for BMI.

### The growth patterns of height and FL in children aged 3–18 years

To analyze this, we calculated the mean, the 10th, 50th, and 90th percentile values (P_10_, P_50_, and P_90_) of height and FL for each age group, separately for boys and girls (Tables 2, 3). Table 2 shows that boys have an average height of 172.2 cm at 14 years old, with a remaining growth potential of approximately 5.4 cm compared to the average height of 177.6 cm at 17 ~ 18 years old. The average FL of boys at 13 years old is 25.2 cm, with less than 1.0 cm of remaining space, compared to the average FL of 25.9 cm at 17 ~ 18 years old.

**Table 2:** Mean, 10th, 50th, and 90th percentile values of height and FL for boys.

Age group (y)	*N*	Height (cm)				FL (cm)			
		Mean ± SD	P_10_	P_50_	P_90_	Mean ± SD	P_10_	P_50_	P_90_
3~	16	102.1 ± 5.1	95.5	101.6	110.0	14.8 ± 1.2	13.0	14.8	16.5
4~	33	109.3 ± 6.1	101.6	107.3	117.4	15.8 ± 1.2	14.5	15.5	17.8
5~	37	115.5 ± 3.8	110.2	116.0	120.5	17.0 ± 1.0	15.5	17.0	18.5
6~	154	120.7 ± 5.0	114.2	121.0	127.8	17.9 ± 1.1	16.5	18.0	19.5
7~	127	129.2 ± 5.8	122.0	129.9	136.3	19.1 ± 1.2	17.5	19.0	20.6
8~	115	134.1 ± 5.8	126.9	134.1	141.9	20.0 ± 1.3	18.5	20.0	22.0
9~	107	139.5 ± 6.7	131.3	139.9	147.7	21.2 ± 1.7	19.0	21.0	23.6
10~	116	145.3 ± 6.3	137.4	145.2	153.8	22.0 ± 1.5	20.0	22.0	24.0
11~	88	153.7 ± 8.4	143.9	152.9	165.1	23.6 ± 1.5	21.5	23.5	25.6
12~	211	161.3 ± 8.7	149.5	161.6	171.9	24.6 ± 1.5	22.5	24.5	26.5
13~	236	167.3 ± 7.0	158.2	167.2	176.4	25.2 ± 1.2	23.5	25.5	26.5
14~	195	172.2 ± 6.6	163.4	172.4	180.7	25.6 ± 1.2	24.0	25.5	27.5
15~	114	174.1 ± 6.3	165.6	175.2	181.9	25.8 ± 1.2	24.0	26.0	27.5
16~	80	175.9 ± 6.5	167.0	175.4	184.0	25.8 ± 1.1	24.1	26.0	27.5
17 ~ 18	113	177.6 ± 6.4	170.0	178.2	185.1	25.9 ± 1.2	24.5	26.0	27.5

The P_10_, P_50_, and P_90_ were the 10th, 50th, and 90th percentile values of height or FL.

**Table 3:** Mean, 10th, 50th, and 90th percentile values of height and FL for girls.

Age group (y)	*N*	Height (cm)				FL (cm)			
		Mean ± SD	P_10_	P_50_	P_90_	Mean ± SD	P_10_	P_50_	P_90_
3~	19	100.9 ± 3.6	95.8	101.3	105.6	14.5 ± 0.7	13.5	14.5	15.5
4~	24	109.3 ± 4.9	104.6	108.5	114.8	15.7 ± 0.8	14.5	15.5	17.0
5~	31	115.4 ± 5.2	108.8	115.7	122.7	16.7 ± 1.2	15.0	16.5	18.4
6~	149	120.8 ± 5.1	114.0	120.8	126.7	17.8 ± 1.0	16.5	18.0	19.0
7~	99	127.8 ± 4.8	121.1	128.0	133.3	18.7 ± 1.1	17.5	19.0	20.0
8~	114	132.9 ± 5.5	126.1	132.6	140.5	19.4 ± 1.2	18.0	19.5	21.0
9~	95	142.0 ± 7.6	131.7	142.1	152.3	21.0 ± 1.5	19.0	21.0	23.0
10~	109	146.2 ± 8.1	135.6	146.0	157.5	21.4 ± 1.6	19.0	21.5	23.5
11~	94	154.6 ± 6.3	146.6	154.2	162.0	22.6 ± 1.2	21.0	22.5	24.3
12~	226	158.1 ± 6.3	150.4	158.4	165.5	23.1 ± 1.1	22.0	23.0	24.5
13~	234	160.8 ± 5.4	153.8	160.6	168.6	23.3 ± 1.0	22.0	23.0	24.5
14~	205	161.4 ± 5.7	154.3	161.5	168.6	23.1 ± 1.1	22.0	23.0	24.5
15~	150	162.3 ± 4.8	155.4	162.6	167.9	23.5 ± 1.1	22.1	23.5	24.5
16~	100	163.7 ± 5.8	156.4	163.0	171.4	23.4 ± 1.1	22.0	23.5	25.0
17 ~ 18	93	163.5 ± 5.7	157.0	162.7	171.2	23.5 ± 0.9	22.0	23.2	24.3

The P_10_, P_50_, and P_90_ were the 10th, 50th, and 90th percentile values of height or FL.

Table 3 shows that girls have an average height of 158.1 cm at 12 years old, with a remaining growth potential of approximately 5.4 cm compared to the average height of 163.5 cm at 17 ~ 18 years old. The average FL of girls at 11 years old is 22.6 cm, with less than 1.0 cm of remaining space, compared to the average FL of 23.5 cm at 17 ~ 18 years old.

**Figure 1:**
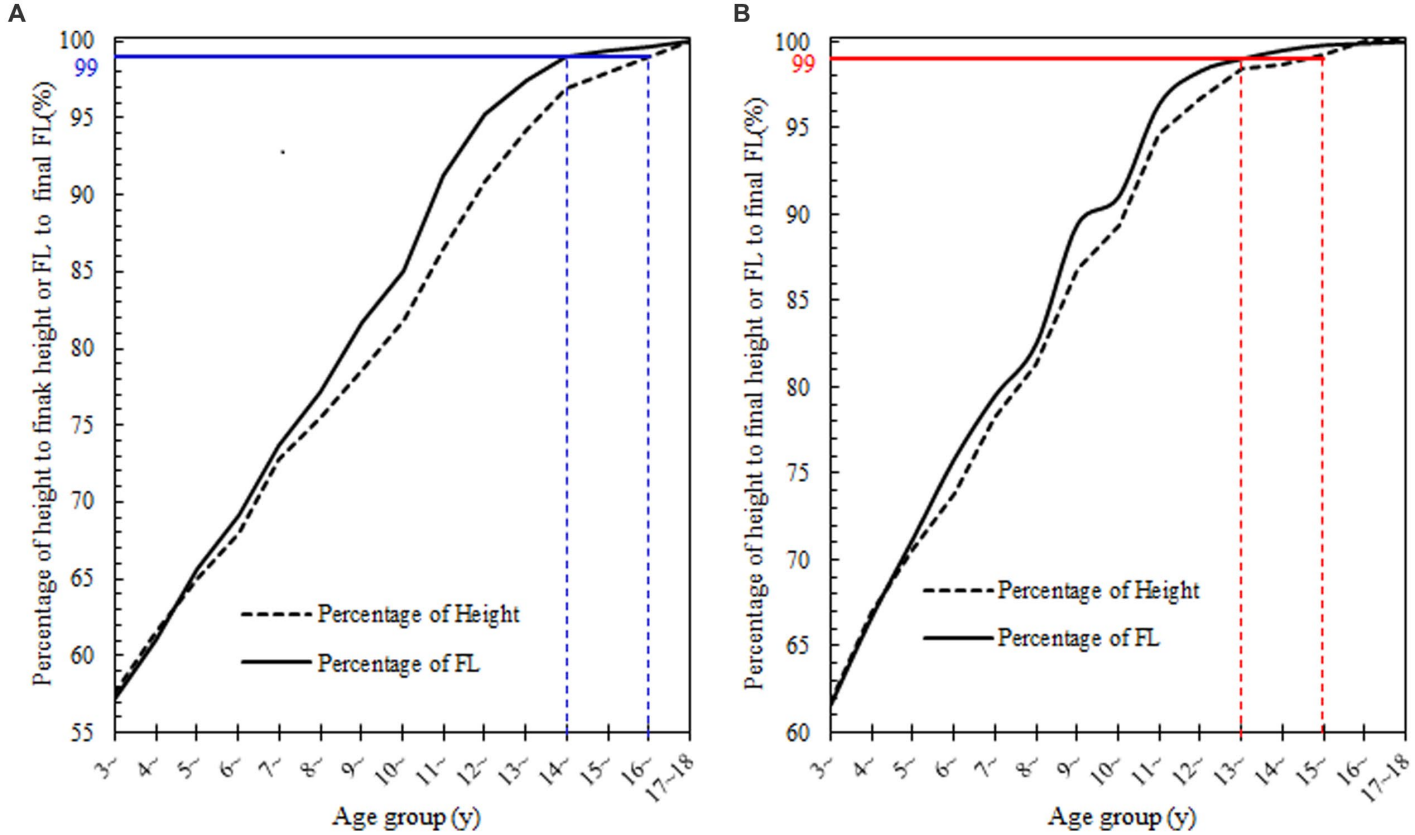
Percentages of height and FL to the final height and FL in different age groups. **(A)** boys and **(B)** girls. In this figure, we further described the growth pattern of height and FL for boys and girls. We can see that FL grows relatively earlier than height, especially after puberty onset, and FL growth ceases 2 years earlier than that of height.

To further analyze the growth patterns of height and FL among different age groups, we calculated the percentages of height and FL to their final height and final FL at each age group. As shown in Figure 1, boys’ height and FL in the 3 ~ age group accounted for approximately 57% of their final height and FL. By the age of 14, their FL had reached over 99% of the final FL, while their height did not reach over 99.0% of the final height until the age of 16. In contrast, girls’ height and FL in the 3 ~ age group accounted for approximately 61.0% of their final height and FL. By the age of 13, their FL had reached over 99.0% of the final FL, while their height did not reach 99.0% of the final height until the age of 15.

### The ages of peak growth for FL and height among children

**Figure 2:**
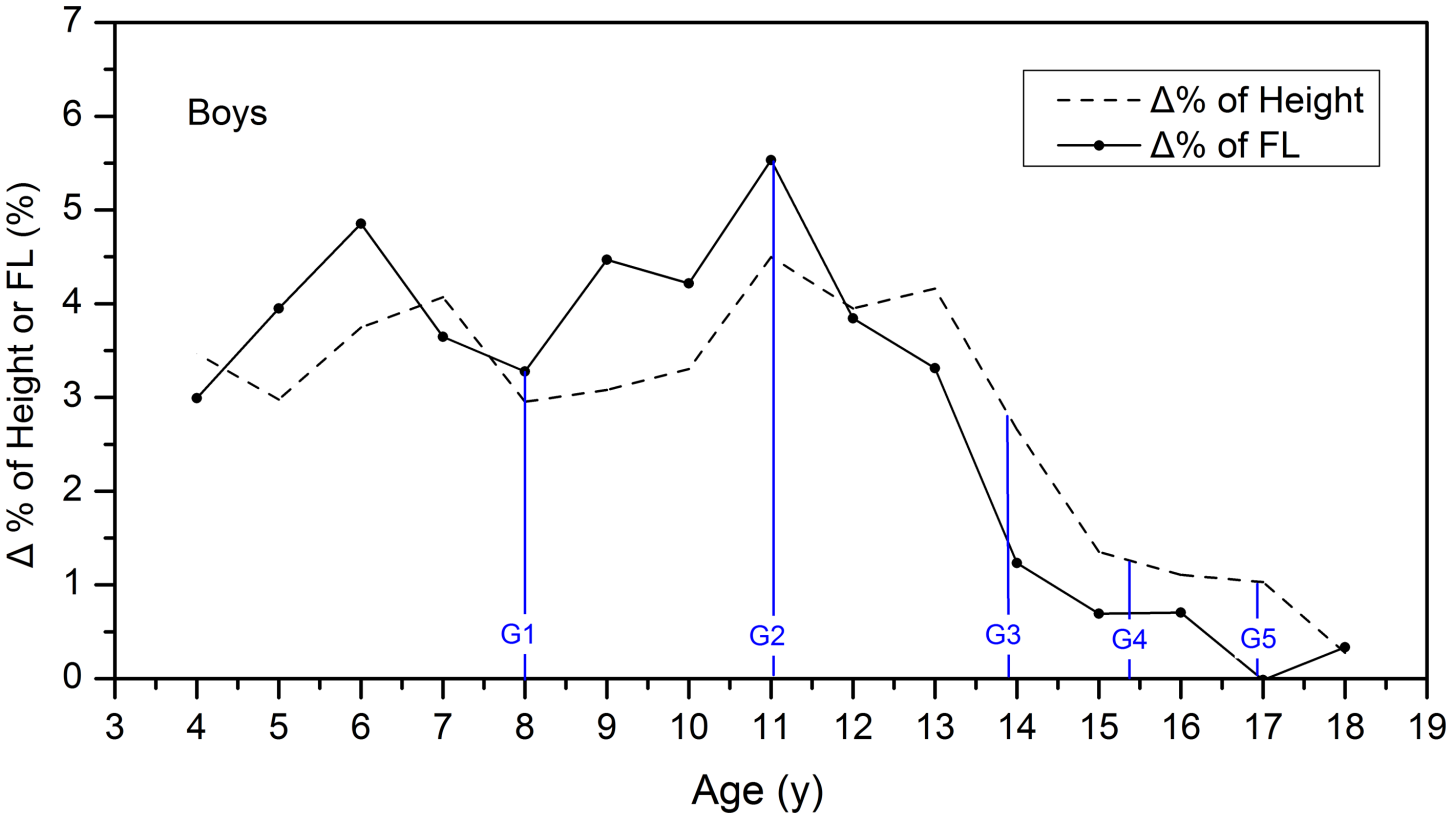
Comparison of the GV of height and FL among boys. Δ % is the increment of percentage values in height and FL per year, which was used to describe and compare the GV of height and FL in different age groups. The blue lines showed the average age of G_1_–G_5_ stages for boys.

There were notable disparities in the GV for height and FL among children. Therefore, it is not advisable to compare actual GV values directly. Instead, GV can be expressed as the increment of percentage values (Δ %) in height and FL per year. Figure 2 reveals that rapid FL growth for girls occurs at 6 and 11 years of age, with the peak growth at 11 years, followed by a sharp decline in GV. Meanwhile, rapid height growth for boys happens between 11 and 13 years of age, with a steep drop in GV after 13 years.

**Figure 3:**
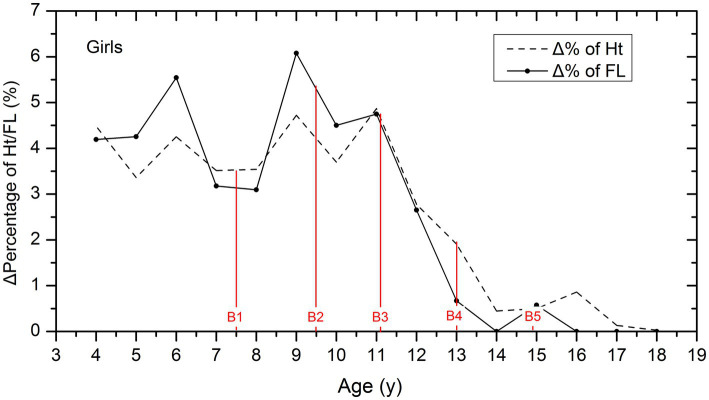
Comparison of the GV of height and FL among girls. Δ % is the increment of percentage values in height and FL per year, which was used to describe and compare the GV of height and FL in different age groups. The red lines showed the average age of B_1_–B_5_ stages for girls.

According to Figure 3, girls experienced the rapid growth of FL at the ages of 6 and 9 years, with the peak velocity at 9 years old. However, the GV gradually declines and drops rapidly after the age of 11 years. As for height, girls experience rapid growth between the ages of 9 and 11 years, with the peak velocity at 11 years old, then the GV declines steeply.

### The association of peak height and FL growth with pubertal development

Table 4 provides data on the age, height, and FL of boys and girls according to their Tanner stages. It is worth noting that girls typically begin puberty earlier than boys, with an onset age of approximately 9.5 years old, which is approximately 2 years earlier than boys.

**Table 4:** Age, height, and FL in different stages of puberty development.

	*N*	Age (y)	Height (cm)	FL (cm)
**Boys**				
G_1_	460	8.0 ± 1.5	130.0 ± 9.5	19.4 ± 1.8
G_2_	365	11.1 ± 1.8	150.4 ± 11.3	22.9 ± 2.0
G_3_	442	13.9 ± 1.3	169.0 ± 7.6	25.4 ± 1.3
G_4_	301	15.3 ± 1.6	173.9 ± 7.0	25.7 ± 1.2
G_5_	64	16.9 ± 0.8	177.0 ± 6.2	26.0 ± 1.2
**Girls**				
B_1_	338	7.5 ± 1.1	126.2 ± 7.3	18.5 ± 1.3
B_2_	116	9.5 ± 1.1	138.9 ± 6.4	20.4 ± 1.4
B_3_	98	11.2 ± 1.3	150.6 ± 8.1	22.2 ± 1.4
B_4_	444	13.0 ± 1.6	158.5 ± 6.6	23.0 ± 1.2
B_5_	652	14.8 ± 1.7	161.7 ± 5.7	23.3 ± 1.0

FL is foot length. G_1_–G_5_ is the Tanner stage of boys based on the degree of penis development and B_1_–B_5_ is the Tanner stage of girls based on the degree of breast development.

For further analysis of the associations of puberty stages with FL and height growth, we marked the average age of the G_1_ to G_5_ stages with the blue lines in Figure 2. We can see that FL growth in boys accelerated before the G_2_ stage, and the peak growth occurred at 11.1 years, which coincides with the average age of the G_2_ stage. In contrast, height growth accelerated after entering the G_2_ stage, and the peak growth occurred between the G_2_ and G_3_ stages.

In addition, for girls, we also marked the average age of B_1_ to B_5_ stages with the red lines in Figure 3, which showed that the peak growth of FL occurred before the B_2_ stage at the age of 9.0. Height growth accelerated after the age of 9, and the peak growth occurred between the B_2_ and B_3_ stages.

## Discussion

The age of this study sample spans from 3 to 18 years old, encompassing the growth and development process from preschool to the end of puberty, which allowed for a more thorough exploration of growth patterns in height and FL and their associations with puberty development. The most recent studies on FL in China have only covered the ages of 7–12 years old and 13–18 years old separately, which are not age-continuous and do not fully reflect the whole growth and development patterns of children (6, 10). Similarly, the latest Spanish study on FL only covers the age range of 3–12 years old, resulting in an incomplete description of growth patterns (4).

From the perspective of age, peak FL growth is earlier than peak height growth, and the age at which FL growth ceases is also approximately 2 years earlier than when height ceases. As shown in Figures 2, 3 of this study, the age of peak FL growth in boys was 11 years old, and in girls, it was 9 years old, which was consistent with the conclusions of previous studies that the age of peak FL growth in girls is 7–9 years old and in boys is 10–11 years old (4, 5, 11, 12). However, the peak height growth in boys occurred between 11 and 13 years old, and 11 years old in girls, which is approximately 2 years later than the peak FL growth. In addition, most studies have shown that the GV of FL in girls slows down after the age of 12–13 years, and in boys, it slows down after the age of 15 (13–15). Figure 1 of this study confirms that girls reach 99% of their final FL at the age of 13, and boys reach 99% of their final FL at the age of 14, which is consistent with previous studies. At the same time, girls reach 99% of their final height at the age of 15, and boys reach 99% of their final height at the age of 16, indicating that the age at which FL growth ceases is approximately 2 years earlier than that of height.

From the perspective of puberty development, peak FL growth occurred around the onset of puberty, and peak height growth occurred between Tanner stages 2 and 3. Peak FL growth can be used as an early predictor of puberty onset (16). This study observed the age of peak FL growth almost coinciding with the mean age of Tanner stage 2. This confirmed Mitra’s conclusion that the peak FL growth is related to the transition from Tanner stages 1 to 2 (5). A cross-sectional study in 2021 also confirmed that the sudden increase in FL growth coincides with the age of Tanner stage 2 (4). While peak height growth occurs later than FL, we observed that the peak height growth occurred between Tanner stages 2 and 3. Although growth patterns may differ among children of different races, the associations between FL growth and puberty stages are generally consistent, and peak FL growth can serve as an early indicator of puberty onset (15, 17).

Additionally, we also identified a small peak in FL growth at the age of 5–6 years old, which is often overlooked. There were limited studies on FL in preschool children, but a few studies have found similar phenomena. Stavlas et al. mentioned that significant changes in foot development occur during the preschool period, as well as during school age and late puberty (18). Other studies also showed that the most active period of foot development is at the age of 6 years old (19, 20). Therefore, it is possible that there is a relatively rapid growth period for FL approximately 6 years when children may not be able to express their needs in a timely and accurate manner. Therefore, parents should pay attention to those children’s FL growth, replace their shoes promptly, and avoid affecting their normal foot growth or causing unnecessary foot injuries and deformities.

### Strengths and limitations

First, this study covers a wide age range from 3 to 18 years old, providing a comprehensive understanding of the growth and development process from preschool to the end of adolescence. This allows for a more accurate reflection of the associations between height, FL growth, and puberty development. Second, this study is limited by its cross-sectional design, which may not accurately capture individual differences in growth and development patterns. This could lead to an underestimation of the peak growth velocity. However, we focused on comparing the growth patterns of height and FL using the same method, which ensures that the results are comparable and can be used as a reference for pediatric clinical practice and healthcare. For further research, we would like to conduct longitudinal follow-up studies to more accurately illustrate the growth pattern of FL in Chinese children.

## Conclusion

Children experience their peak FL growth at 9 years old for girls and 11 years old for boys, which were both 2 years earlier than the peak height growth. While peak FL growth occurs around the onset of puberty, peak height growth typically occurs between Tanner stages 2 and 3. By monitoring the GV of FL, height, and pubertal stages, it is possible to identify any potential complications in children’s growth and development early and intervene promptly.

## Data availability statement

The raw data supporting the conclusions of this article will be made available by the authors, without undue reservation.

## Ethics statement

The studies involving humans were approved by Local Ethics Committee at Capital Institute of Pediatrics, Beijing. The studies were conducted in accordance with the local legislation and institutional requirements. Written informed consent for participation in this study was provided by the participants’ legal guardians/next of kin.

## Author contributions

H-HW: Conceptualization, Data curation, Formal analysis, Investigation, Methodology, Software, Validation, Writing – original draft, Writing – review & editing. Y-QZ: Data curation, Investigation, Methodology, Supervision, Writing – original draft. C-DY: Data curation, Investigation, Methodology, Supervision, Writing – original draft. LY: Data curation, Investigation, Supervision, Writing – review & editing. CT: Data curation, Investigation, Methodology, Supervision, Writing – review & editing. WS: Data curation, Investigation, Methodology, Writing – original draft. TL: Funding acquisition, Investigation, Software, Supervision, Writing – review & editing. GH: Data curation, Investigation, Supervision, Writing – review & editing. DH: Data curation, Investigation, Software, Supervision, Writing – review & editing. F-FC: Conceptualization, Investigation, Resources, Supervision, Visualization, Writing – review & editing. J-TL: Conceptualization, Funding acquisition, Investigation, Resources, Supervision, Visualization, Writing – review & editing. S-lL: Funding acquisition, Resources, Supervision, Visualization, Writing – review & editing. X-NZ: Conceptualization, Data curation, Funding acquisition, Investigation, Methodology, Project administration, Resources, Software, Supervision, Validation, Visualization, Writing – review & editing.
